# Lithium samarium polyphosphate, LiSm(PO_3_)_4_

**DOI:** 10.1107/S1600536809055822

**Published:** 2010-01-09

**Authors:** Dan Zhao, Feifei Li, Wendan Cheng, Hao Zhang

**Affiliations:** a Department of Physics and Chemistry, Henan Polytechnic University, Jiaozuo, Henan 454000, People’s Republic of China; b State Key Laboratory of Structural Chemistry, Fujian Institute of Research on the Structure of Matter, Chinese Academy of Sciences, Fuzhou, Fujian 350002, People’s Republic of China

## Abstract

The mixed-metal rare-earth polyphosphate LiSm(PO_3_)_4_ consists of a three-dimensional framework in which zigzag [(PO_3_)_*n*_]^*n*−^ chains with a periodicity of four PO_4_ tetrahedra are connected through Li^+^ and Sm^3+^ ions (both with 2. symmetry).

## Related literature

For the structures, properties and applications of condensed alkaline metal–rare earth polyphosphates with the general formula *MLn*(PO_3_)_4_ (*M* = alkali metal, *Ln* = rare earth metal), see: Ferid *et al.* (1984 ▶); Ettis *et al.* (2003 ▶); Parreu *et al.* (2007 ▶); Zhu *et al.* (2007 ▶); Ben Zarkouna *et al.* (2007 ▶).

## Experimental

### 

#### Crystal data

LiSm(PO_3_)_4_
*M* *_r_* = 473.17
Monoclinic, 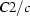

*a* = 16.379 (2) Å
*b* = 7.0499 (9) Å
*c* = 9.6936 (12) Å
β = 126.138 (2)°
*V* = 903.96 (19) Å^3^
*Z* = 4
Mo *K*α radiation
μ = 7.27 mm^−1^
*T* = 298 K
0.20 × 0.15 × 0.05 mm

#### Data collection

Bruker SMART CCD area-detector diffractometer
Absorption correction: multi-scan (*SADABS*; Bruker, 1997 ▶) *T*_min_ = 0.439, *T*_max_ = 1.000
2405 measured reflections
854 independent reflections
843 reflections with *I* > 2σ(*I*)
*R*_int_ = 0.023

#### Refinement

*R*[*F* ^2^ > 2σ(*F* ^2^)] = 0.019
*wR*(*F* ^2^) = 0.048
*S* = 1.09
854 reflections
84 parameters
Δρ_max_ = 1.02 e Å^−3^
Δρ_min_ = −0.71 e Å^−3^

### 

Data collection: *SMART* (Bruker, 1997 ▶); cell refinement: *SAINT* (Bruker, 1997 ▶); data reduction: *SAINT*; program(s) used to solve structure: *SHELXS97* (Sheldrick, 2008 ▶); program(s) used to refine structure: *SHELXL97* (Sheldrick, 2008 ▶); molecular graphics: *SHELXTL* (Sheldrick, 2008 ▶) and *PLATON* (Spek, 2009 ▶); software used to prepare material for publication: *SHELXTL*.

## Supplementary Material

Crystal structure: contains datablocks I, global. DOI: 10.1107/S1600536809055822/mg2086sup1.cif

Structure factors: contains datablocks I. DOI: 10.1107/S1600536809055822/mg2086Isup2.hkl

Additional supplementary materials:  crystallographic information; 3D view; checkCIF report

## Figures and Tables

**Fig. 1.**
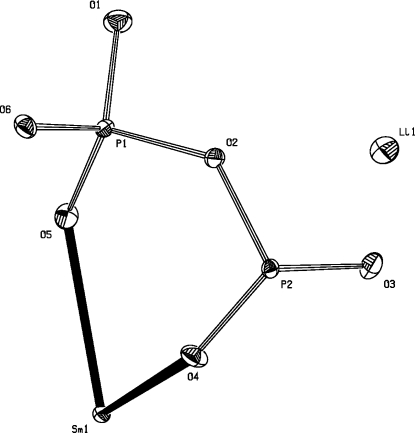
Part of the structure of LiSm(PO3)4 showing the labelling of all atoms. Displacement ellipsoids are drawn at the 50% probability level.

**Fig. 2.**
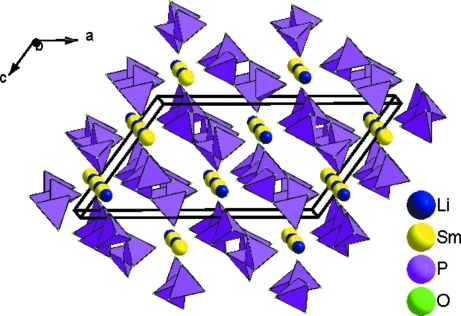
Projection of the structure of LiSm(PO3)4 down the b axis.

## supplementary crystallographic information

### Comment

Interest in alkali-metal rare-earth polyphosphates stems from their physical properties, such as high luminescence efficiency (Ettis *et al.*, 2003; Parreu *et al.*, 2007; Zhu *et al.*, 2007). The compound LiSm(PO_3_)_4_ has been reported but only unit cell parameters have been refined from powder X-ray diffraction data (Ferid *et al.*, 1984). The single-crystal structure determination performed here confirms that it is isotypic with LiLn(PO_3_)_4_ (Ln = Y, La, Nd, Eu, Gd, Tb, Dy, Er, Yb) (Ben Zarkouna *et al.*, 2007). The structure features two P sites (Fig. 1) centred within PO_4_ tetrahedra, which share common corners (O2 or O6) to form infinite zigzag chains (PO_3_)_n_^n-^ that are aligned parallel to the *b*-direction and are linked together by four-coordinate Li^+^ and eight-coordinate Sm^3+^ ions (Fig. 2).

### Experimental

Finely ground reagents Li_2_CO_3_, Sm_2_O_3_, and NH_4_H_2_PO_4_ were mixed in a molar ratio of Li:Sm:P = 7:1:10, placed in a Pt crucible, and heated at 673 K for 4 h. The mixture was reground and heated at 1073 K for 20 h, cooled to 873 K at a rate of 4 K h^-1^, and then quenched to room temperature. A few yellow prism-shaped crystals of the title compound were obtained.

### Refinement

The highest peak and the deepest hole in the difference electron density map are located 0.92 Å and 1.11 Å, respectively, from Sm1.

### Crystal data

**Table:**

LiSm(PO_3_)_4_	*F*(000) = 884
*M**_r_* = 473.17	*D*_x_ = 3.477 Mg m^−^^3^
Monoclinic, *C*2/*c*	Mo *K*α radiation, λ = 0.71073 Å
Hall symbol: -C 2yc	Cell parameters from 487 reflections
*a* = 16.379 (2) Å	θ = 2.1–23.0°
*b* = 7.0499 (9) Å	µ = 7.27 mm^−^^1^
*c* = 9.6936 (12) Å	*T* = 298 K
β = 126.138 (2)°	Prism, yellow
*V* = 903.96 (19) Å^3^	0.20 × 0.15 × 0.05 mm
*Z* = 4	

### Data collection

**Table:**

Bruker SMART CCD area-detector diffractometer	854 independent reflections
Radiation source: fine-focus sealed tube	843 reflections with *I* > 2σ(*I*)
graphite	*R*_int_ = 0.023
φ and ω scans	θ_max_ = 25.7°, θ_min_ = 3.1°
Absorption correction: multi-scan (*SADABS*; Bruker, 1997)	*h* = −20→19
*T*_min_ = 0.439, *T*_max_ = 1.000	*k* = −8→8
2405 measured reflections	*l* = −10→11

### Refinement

**Table:**

Refinement on *F*^2^	Primary atom site location: structure-invariant direct methods
Least-squares matrix: full	Secondary atom site location: difference Fourier map
*R*[*F*^2^ > 2σ(*F*^2^)] = 0.019	*w* = 1/[σ^2^(*F*_o_^2^) + (0.0274*P*)^2^ + 6.0112*P*] where *P* = (*F*_o_^2^ + 2*F*_c_^2^)/3
*wR*(*F*^2^) = 0.048	(Δ/σ)_max_ = 0.001
*S* = 1.09	Δρ_max_ = 1.02 e Å^−^^3^
854 reflections	Δρ_min_ = −0.71 e Å^−^^3^
84 parameters	Extinction correction: *SHELXL97* (Sheldrick, 2008), Fc^*^=kFc[1+0.001xFc^2^λ^3^/sin(2θ)]^-1/4^
0 restraints	Extinction coefficient: 0.0069 (3)

### Special details

**Table:**

Geometry. All e.s.d.'s (except the e.s.d. in the dihedral angle between two l.s. planes) are estimated using the full covariance matrix. The cell e.s.d.'s are taken into account individually in the estimation of e.s.d.'s in distances, angles and torsion angles; correlations between e.s.d.'s in cell parameters are only used when they are defined by crystal symmetry. An approximate (isotropic) treatment of cell e.s.d.'s is used for estimating e.s.d.'s involving l.s. planes.
Refinement. Refinement of *F*^2^ against ALL reflections. The weighted *R*-factor *wR* and goodness of fit *S* are based on *F*^2^, conventional *R*-factors *R* are based on *F*, with *F* set to zero for negative *F*^2^. The threshold expression of *F*^2^ > σ(*F*^2^) is used only for calculating *R*-factors(gt) *etc*. and is not relevant to the choice of reflections for refinement. *R*-factors based on *F*^2^ are statistically about twice as large as those based on *F*, and *R*- factors based on ALL data will be even larger.

### Fractional atomic coordinates and isotropic or equivalent isotropic displacement parameters (Å^2^)

**Table:**

	*x*	*y*	*z*	*U*_iso_*/*U*_eq_	
Li1	0.5000	0.2975 (12)	0.7500	0.014 (2)	
Sm1	0.5000	0.20102 (3)	0.2500	0.00541 (15)	
P1	0.36163 (7)	0.55515 (13)	0.33744 (11)	0.0057 (2)	
P2	0.35188 (7)	0.15529 (14)	0.40335 (12)	0.0056 (2)	
O1	0.3857 (2)	0.7182 (4)	0.4524 (4)	0.0117 (6)	
O2	0.3410 (2)	0.3787 (4)	0.4149 (3)	0.0094 (5)	
O3	0.4267 (2)	0.0930 (4)	0.5830 (3)	0.0104 (5)	
O4	0.3705 (2)	0.1147 (4)	0.2737 (3)	0.0104 (5)	
O5	0.43430 (19)	0.5038 (4)	0.2978 (3)	0.0094 (5)	
O6	0.25564 (19)	0.5836 (4)	0.1557 (3)	0.0091 (5)	

### Atomic displacement parameters (Å^2^)

**Table:**

	*U*^11^	*U*^22^	*U*^33^	*U*^12^	*U*^13^	*U*^23^
Li1	0.018 (5)	0.011 (5)	0.016 (5)	0.000	0.011 (5)	0.000
Sm1	0.00650 (19)	0.00499 (19)	0.00591 (19)	0.000	0.00431 (14)	0.000
P1	0.0060 (4)	0.0046 (4)	0.0068 (5)	0.0004 (3)	0.0040 (4)	0.0004 (3)
P2	0.0059 (4)	0.0053 (4)	0.0062 (5)	−0.0005 (3)	0.0040 (4)	0.0004 (4)
O1	0.0130 (14)	0.0093 (14)	0.0112 (15)	−0.0007 (10)	0.0063 (13)	−0.0026 (10)
O2	0.0167 (13)	0.0052 (13)	0.0133 (13)	0.0000 (10)	0.0126 (12)	0.0009 (10)
O3	0.0108 (13)	0.0076 (12)	0.0094 (13)	0.0003 (10)	0.0041 (11)	0.0016 (10)
O4	0.0127 (13)	0.0110 (13)	0.0126 (13)	−0.0019 (11)	0.0102 (11)	−0.0026 (11)
O5	0.0076 (12)	0.0107 (13)	0.0111 (13)	0.0012 (10)	0.0061 (11)	0.0021 (10)
O6	0.0083 (12)	0.0125 (13)	0.0073 (12)	0.0027 (10)	0.0049 (11)	0.0012 (10)

### Geometric parameters (Å, °)

**Table:**

Li1—O3	1.962 (7)	Sm1—O5^iv^	2.553 (3)
Li1—O3^i^	1.962 (7)	P1—O1	1.483 (3)
Li1—O5^ii^	1.980 (7)	P1—O5	1.495 (3)
Li1—O5^iii^	1.980 (7)	P1—O2	1.590 (3)
Li1—P2	2.927 (3)	P1—O6	1.597 (3)
Li1—P2^i^	2.927 (3)	P1—Li1^iii^	3.033 (3)
Li1—P1^ii^	3.033 (3)	P2—O4	1.485 (3)
Li1—P1^iii^	3.033 (3)	P2—O3	1.487 (3)
Sm1—O4	2.345 (3)	P2—O6^viii^	1.580 (3)
Sm1—O4^iv^	2.345 (3)	P2—O2	1.596 (3)
Sm1—O1^iii^	2.405 (3)	O1—Sm1^iii^	2.405 (3)
Sm1—O1^v^	2.405 (3)	O3—Sm1^vii^	2.463 (3)
Sm1—O3^vi^	2.463 (3)	O5—Li1^iii^	1.980 (7)
Sm1—O3^vii^	2.463 (3)	O6—P2^ix^	1.580 (3)
Sm1—O5	2.553 (3)		
			
O3—Li1—O3^i^	85.4 (4)	O3^vi^—Sm1—O3^vii^	65.38 (12)
O3—Li1—O5^ii^	124.08 (11)	O4—Sm1—O5	72.34 (9)
O3^i^—Li1—O5^ii^	118.63 (11)	O4^iv^—Sm1—O5	137.49 (9)
O3—Li1—O5^iii^	118.63 (11)	O1^iii^—Sm1—O5	72.38 (9)
O3^i^—Li1—O5^iii^	124.08 (11)	O1^v^—Sm1—O5	84.63 (9)
O5^ii^—Li1—O5^iii^	90.0 (4)	O3^vi^—Sm1—O5	136.79 (8)
O3—Li1—P2	27.29 (8)	O3^vii^—Sm1—O5	132.74 (9)
O3^i^—Li1—P2	112.7 (3)	O4—Sm1—O5^iv^	137.49 (9)
O5^ii^—Li1—P2	108.29 (11)	O4^iv^—Sm1—O5^iv^	72.34 (9)
O5^iii^—Li1—P2	99.83 (10)	O1^iii^—Sm1—O5^iv^	84.63 (9)
O3—Li1—P2^i^	112.7 (3)	O1^v^—Sm1—O5^iv^	72.38 (9)
O3^i^—Li1—P2^i^	27.29 (8)	O3^vi^—Sm1—O5^iv^	132.74 (9)
O5^ii^—Li1—P2^i^	99.83 (10)	O3^vii^—Sm1—O5^iv^	136.79 (8)
O5^iii^—Li1—P2^i^	108.29 (11)	O5—Sm1—O5^iv^	66.51 (12)
P2—Li1—P2^i^	139.9 (3)	O4—Sm1—Li1^vii^	74.97 (7)
O3—Li1—P1^ii^	106.74 (11)	O4^iv^—Sm1—Li1^vii^	74.97 (7)
O3^i^—Li1—P1^ii^	102.43 (10)	O1^iii^—Sm1—Li1^vii^	103.71 (6)
O5^ii^—Li1—P1^ii^	25.02 (8)	O1^v^—Sm1—Li1^vii^	103.71 (6)
O5^iii^—Li1—P1^ii^	114.9 (3)	O3^vi^—Sm1—Li1^vii^	32.69 (6)
P2—Li1—P1^ii^	100.84 (3)	O3^vii^—Sm1—Li1^vii^	32.69 (6)
P2^i^—Li1—P1^ii^	92.67 (3)	O5—Sm1—Li1^vii^	146.74 (6)
O3—Li1—P1^iii^	102.43 (10)	O5^iv^—Sm1—Li1^vii^	146.74 (6)
O3^i^—Li1—P1^iii^	106.74 (11)	O4—Sm1—Li1^iii^	105.03 (7)
O5^ii^—Li1—P1^iii^	114.9 (3)	O4^iv^—Sm1—Li1^iii^	105.03 (7)
O5^iii^—Li1—P1^iii^	25.02 (8)	O1^iii^—Sm1—Li1^iii^	76.29 (6)
P2—Li1—P1^iii^	92.67 (3)	O1^v^—Sm1—Li1^iii^	76.29 (6)
P2^i^—Li1—P1^iii^	100.84 (3)	O3^vi^—Sm1—Li1^iii^	147.31 (6)
P1^ii^—Li1—P1^iii^	139.9 (3)	O3^vii^—Sm1—Li1^iii^	147.31 (6)
O3—Li1—Sm1^vii^	42.70 (19)	O5—Sm1—Li1^iii^	33.26 (6)
O3^i^—Li1—Sm1^vii^	42.70 (19)	O5^iv^—Sm1—Li1^iii^	33.26 (6)
O5^ii^—Li1—Sm1^vii^	135.01 (19)	Li1^vii^—Sm1—Li1^iii^	180.000 (1)
O5^iii^—Li1—Sm1^vii^	135.01 (19)	O1—P1—O5	118.76 (17)
P2—Li1—Sm1^vii^	69.97 (16)	O1—P1—O2	106.74 (15)
P2^i^—Li1—Sm1^vii^	69.97 (16)	O5—P1—O2	110.85 (15)
P1^ii^—Li1—Sm1^vii^	110.03 (16)	O1—P1—O6	111.48 (16)
P1^iii^—Li1—Sm1^vii^	110.03 (16)	O5—P1—O6	105.01 (14)
O3—Li1—Sm1^iii^	137.30 (19)	O2—P1—O6	102.92 (15)
O3^i^—Li1—Sm1^iii^	137.30 (19)	O1—P1—Li1^iii^	91.88 (18)
O5^ii^—Li1—Sm1^iii^	44.99 (19)	O2—P1—Li1^iii^	143.38 (18)
O5^iii^—Li1—Sm1^iii^	44.99 (19)	O6—P1—Li1^iii^	98.86 (11)
P2—Li1—Sm1^iii^	110.03 (16)	O4—P2—O3	119.71 (16)
P2^i^—Li1—Sm1^iii^	110.03 (16)	O4—P2—O6^viii^	111.89 (15)
P1^ii^—Li1—Sm1^iii^	69.97 (16)	O3—P2—O6^viii^	107.63 (15)
P1^iii^—Li1—Sm1^iii^	69.97 (16)	O4—P2—O2	109.68 (15)
Sm1^vii^—Li1—Sm1^iii^	180.0	O3—P2—O2	104.96 (15)
O4—Sm1—O4^iv^	149.93 (13)	O6^viii^—P2—O2	101.19 (15)
O4—Sm1—O1^iii^	93.04 (10)	O4—P2—Li1	126.36 (11)
O4^iv^—Sm1—O1^iii^	94.01 (10)	O6^viii^—P2—Li1	121.18 (11)
O4—Sm1—O1^v^	94.01 (10)	O2—P2—Li1	68.45 (19)
O4^iv^—Sm1—O1^v^	93.04 (10)	P1—O1—Sm1^iii^	139.38 (17)
O1^iii^—Sm1—O1^v^	152.59 (13)	P1—O2—P2	132.45 (17)
O4—Sm1—O3^vi^	74.20 (9)	P2—O3—Li1	115.5 (2)
O4^iv^—Sm1—O3^vi^	80.54 (9)	P2—O3—Sm1^vii^	139.82 (16)
O1^iii^—Sm1—O3^vi^	136.12 (9)	Li1—O3—Sm1^vii^	104.6 (2)
O1^v^—Sm1—O3^vi^	71.22 (9)	P2—O4—Sm1	132.98 (16)
O4—Sm1—O3^vii^	80.54 (9)	P1—O5—Li1^iii^	120.9 (2)
O4^iv^—Sm1—O3^vii^	74.20 (9)	P1—O5—Sm1	137.19 (16)
O1^iii^—Sm1—O3^vii^	71.22 (9)	Li1^iii^—O5—Sm1	101.8 (2)
O1^v^—Sm1—O3^vii^	136.12 (9)	P2^ix^—O6—P1	133.96 (18)

Symmetry codes: (i) −*x*+1, *y*, −*z*+3/2; (ii) *x*, −*y*+1, *z*+1/2; (iii) −*x*+1, −*y*+1, −*z*+1; (iv) −*x*+1, *y*, −*z*+1/2; (v) *x*, −*y*+1, *z*−1/2; (vi) *x*, −*y*, *z*−1/2; (vii) −*x*+1, −*y*, −*z*+1; (viii) −*x*+1/2, *y*−1/2, −*z*+1/2; (ix) −*x*+1/2, *y*+1/2, −*z*+1/2.
